# Evolution of neural circuitry and cognition

**DOI:** 10.1098/rsbl.2023.0576

**Published:** 2024-05-15

**Authors:** Max S. Farnworth, Stephen H. Montgomery

**Affiliations:** ^1^ School of Biological Sciences, University of Bristol, Bristol, UK

**Keywords:** brain evolution, evolvability, mushroom body, central complex, *Heliconius*

## Abstract

Neural circuits govern the interface between the external environment, internal cues and outwardly directed behaviours. To process multiple environmental stimuli and integrate these with internal state requires considerable neural computation. Expansion in neural network size, most readily represented by whole brain size, has historically been linked to behavioural complexity, or the predominance of cognitive behaviours. Yet, it is largely unclear which aspects of circuit variation impact variation in performance. A key question in the field of evolutionary neurobiology is therefore how neural circuits evolve to allow improved behavioural performance or innovation. We discuss this question by first exploring how volumetric changes in brain areas reflect actual neural circuit change. We explore three major axes of neural circuit evolution—replication, restructuring and reconditioning of cells and circuits—and discuss how these could relate to broader phenotypes and behavioural variation. This discussion touches on the relevant uses and limitations of volumetrics, while advocating a more circuit-based view of cognition. We then use this framework to showcase an example from the insect brain, the multi-sensory integration and internal processing that is shared between the mushroom bodies and central complex. We end by identifying future trends in this research area, which promise to advance the field of evolutionary neurobiology.

## 1. Introduction

Behaviour largely stems from the brain, a complex network of neural cells connected to form circuits that integrate external sensory stimuli with internal state cues to produce a context-dependent behavioural response. Evolutionary processes act on variation in these behavioural outcomes rather than the neural traits that underpin them, but the adaptive landscape of behaviour will likely be shaped in part by functional variation, costs and constraints imposed on neural traits. To establish, and understand, a causal link between behavioural evolution and changes in neural circuits is a major challenge. Historically, we have often used anatomical proxies to infer changes in the make-up of neural circuits. Specifically, the volume, mass or more recently cell number, of whole brains or anatomical structures, have been used to draw inferences about the evolutionary processes shaping brains and behaviour [1–4]. This comparative approach has provided insights into the co-evolution of neural structures [5–10], associations with ecological or behavioural traits [11–18] and dominant constraints on neural investment [19–24]. Brain size, in particular, has long been linked to the idea of cognition, or specifically biological intelligence, as ‘a dimension of information processing capacity’ for which size can be an ‘estimator of neural parameters likely to be correlated with total information processing capacity’ [25]. Indeed, in specific circumstances, volumes of sub-regions can be extremely informative, because the underlying local circuitry is modified throughout evolution by modifying parts that are closely reflected in volumetric changes [26]. As we discuss below, every sub-region still ultimately needs to be placed in the context of its circuitry [3,26]. Hence, despite extensive efforts to use more refined metrics [27–29], the links between brain size and cognition often remain ambiguous [3,26,30–32] and focus on variation in proxies inherently limits the resolution at which both evolutionary and neural processes can be understood [33].

An alternative approach has been a narrower focus on select species, to investigate evolutionary processes through the understanding of isolated but highly detailed examinations of fine anatomical differences. Recently, it has become accessible to go beyond measures of size and cellular investment to identify specific neural circuit changes that underlie behavioural differences [34–39]. Indeed, whole or partial brain connectomes increasingly underpin revolutionary advancements in *Drosophila* neuroscience where their combination with genetic tools is particularly powerful [40–43]. However, in an increasing array of insects [44,45], and at a coarser level of vertebrates [46–50], we have the foundations for a global and accessible anatomical description of neural circuits. To incorporate such labour and computationally intensive data into phylogenetically rich taxon sampling is a major endeavour, but one that must be undertaken to understand behavioural diversity, as neural circuitry remains the closest structural and functional guide to cognitive evolution.

With this review, we explore the relationship between circuit changes in the neural make-up of the brain, and discuss potential differences in their behavioural relevance. We aim to highlight how considering circuit variation can contextualize volumetric variation, and how different axes of neural variation may be linked to specific modes of behavioural change.

## 2. Towards a circuit-based view of cognition

**Figure 1.**
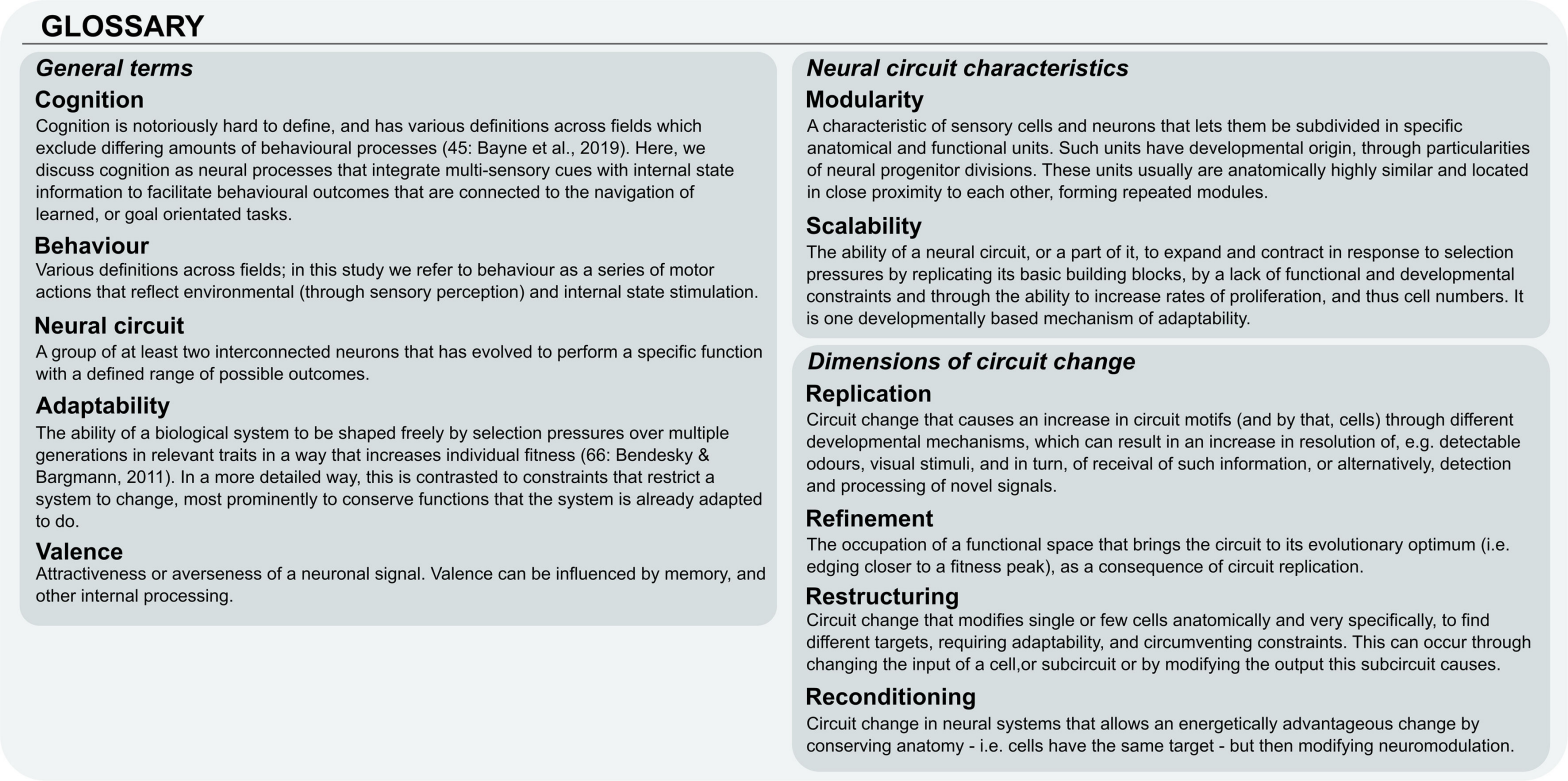
Glossary.

Definitions of cognition, and the behaviours invoked when discussing it, vary from those that encompass wide-ranging processes, to those that reserve cognition for specific processing categories [51,52]. However, selection shapes behaviour within the bounds of a common evolutionary process and a finite range of perceptible environments. As such, definitions of cognition are most useful when they encapsulate processes that enable comparisons across species. These often focus on precise processes, but are not tied to species-specific motivation or behavioural outcomes [53]. As such, identifying specific neural mechanisms that support such functions is a critical step [54]. These mechanisms may, for example, allow for internal representation of the external world, to support flexible, rather than stereotypical, reactions to environmental cues (figure 1).

**Figure 2.**
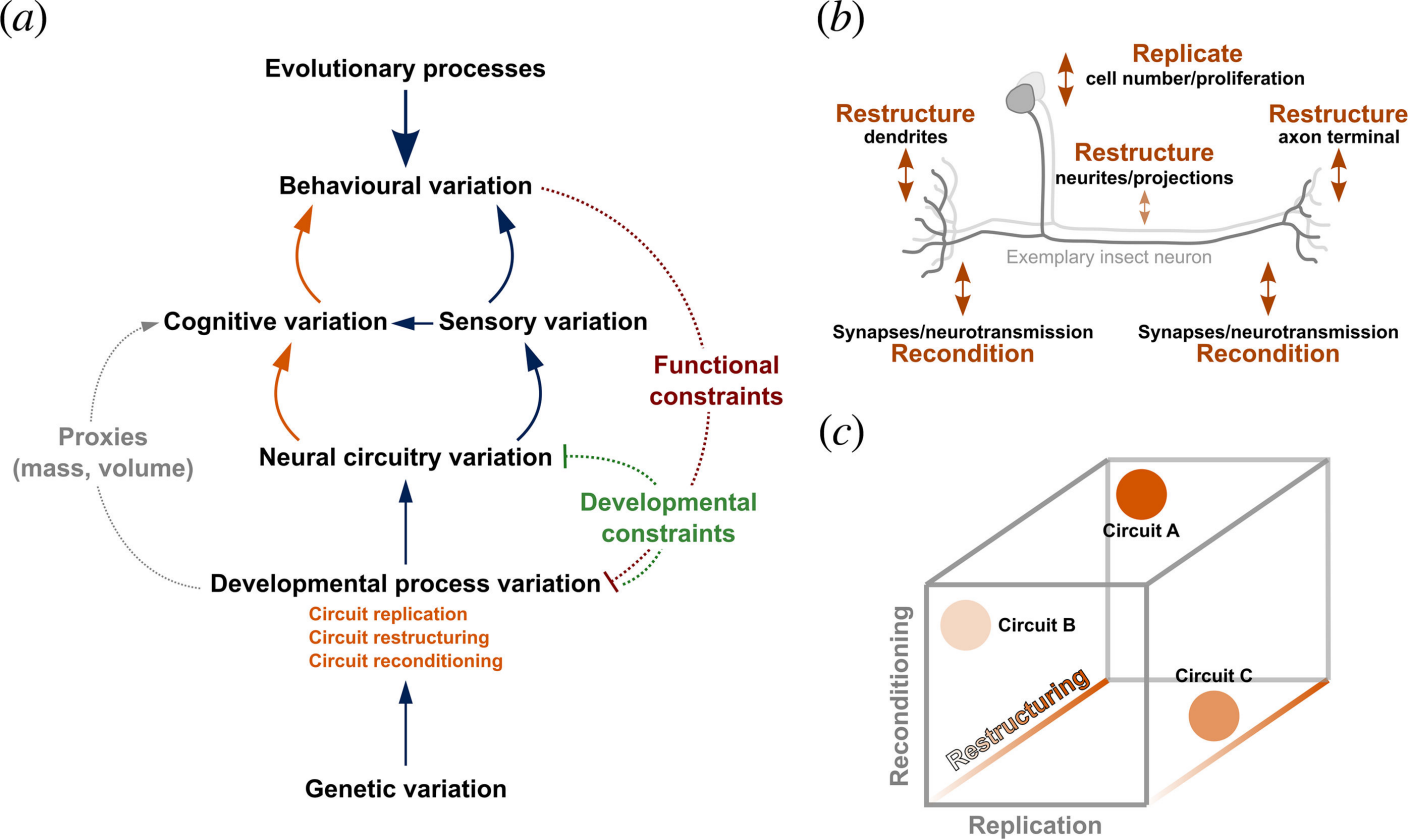
Conceptual framework of the circuit-based view of cognition. (***a***) Hierarchy of terms and concepts that frame the focus of this work, with key areas of discussion in orange. Arrows with solid lines indicate causative links. Additional lines indicate alternative measures and important constraints on variation. (***b***) Illustration of observed evolutionary changes on an exemplary insect neuron. (***c***) Three-dimensional space onto which each sub-circuit can be placed, defined by three axes of circuit change.

Here, we favour a neural circuit-based view of cognition, which focuses on specific neural functions that are evolutionary malleable, and therefore capable of facilitating behavioural innovation and modification (figure *2a*). We distinguish processes that solely involve perception and parcellation of sensory information from those that are integrative, by combining internal states (e.g. memory) with sensory stimuli, to produce a behavioural output [51,55] (figure *2a*). Such separation does not imply a hierarchical organization of cognitive behaviour, or the presence of non-cognitive neural processes, as ultimately all behaviour relies on perception, integration and response to sensory stimuli. However, a critical conceptual distinction is that the selection regimes that shape different phases of information flow can differ, despite the functional non-independence of peripheral and central circuits. For example, peripheral sensory structures and pathways must ensure that adequate and appropriate environmental stimuli are captured and filtered from background signals (i.e. perception and perceptual processing) for a species’ behavioural repertoire, which will be optimized independently from downstream use of that sensory information. While good examples exist of neural change in sensory perception circuits being linked to specific behaviours [36–38,56], the evolution of circuits that support integrative tasks, such as memory or motivation-dependent action, are less frequently examined. It is these downstream processes that we focus on here, and which are most often invoked in studies linking brain size and cognition.

## 3. Mechanisms of neural circuit evolution

To understand the functional links between cognitively guided behaviour and the architecture of neural circuits, an understanding of how neural circuits and their components can accommodate cognitive evolution is essential. Here, we focus on three major axes of neural change, which we term circuit replication, restructuring and reconditioning (figures 1 and 2).

### 3.1. Circuit replication

*Circuit replication*—from duplication to more extensive expansions—causes an increase in circuit motifs (and by necessity, cells), and is often invoked in studies of brain expansion. For example, circuits involved in avian song production [57,58] and electroreception in mormyrid fishes [17] likely involved circuit replication, while a specific conserved circuit motif is duplicated during cerebellar expansion in vertebrates [59]. As part of duplicated circuit motifs, increased populations of specific cell types may influence cognitive performance by either occupying novel or conserved functional space within the larger circuit. The occupation of a functional space that brings a circuit closer to an evolutionary optimum (i.e. closer to a fitness peak) is what we refer to as functional refinement. Circuit duplication offers particular opportunity for change through refinement. As the original circuit is conserved and functional, duplicated cells and circuit motifs have greater scope to circumvent any functional constraints placed on the original circuit [56,60,61]. This is the case, for example, in pheromone-tuned circuitry in *Drosophila*, where parallel morphologies after duplication allow for retuning to a novel stimulus [56]. Alternatively, rather than sensitivity to a novel stimulus, replication and refinement can result in an increase in resolution. For example, genetic perturbance experiments in *Drosophila* demonstrate that an increase in Kenyon cells, the internal cell type of the integrative mushroom bodies, can lead to an increased resolution of odour concepts [62]. Where natural variation exists in cell production within a population, this may therefore directly lead to variation in neural processing performance.

Circuits composed of small numbers of cell types, with shared developmental origins should theoretically be particularly open to evolutionary expansion and contraction. First, circuits with fewer cell types likely have more simple, repeated motifs, suggesting the function is already scalable. Second, developmentally, modifying the number of proliferative cycles of progenitor cells [63–67] can rapidly increase or decrease cell number. Even where circuit motifs require multiple developmentally distinct cell types, motif number may be controlled through shared genetic regulation of motif components, or through selection to maintain co-evolutionary relationships of multiple cell type abundance. For example, across drosophilid species, circuit replication of olfactory sensory neurons is implicated in variation in sensory ecology while upstream projection and integrative neurons are highly conserved, but with specific morphological and physiological modifications [37,38,68–70].

### 3.2. Circuit restructuring

*Restructuring* of circuits occurs through anatomical modifications of a single or a few cells, altering specific connections (figure *2b*). By altering patterns of connections between sensory and downstream circuits, a key functional implication is that behavioural evolution can occur through co-option of existing circuits for new contexts, meaning different cues or combinations of cues may be used in common behavioural processes across species. For example, as discussed below, the mushroom body circuitry of insects has been repeatedly remodelled for processing predominantly visual or olfactory information, or for combinations of cues. These changes reflect the quality of information provided by each sensory domain given a species’ particular niche [71,72].

Developmentally, circuits can be restructured in this way by changes at cellular regions involved in signal transduction—axons, neurites, dendrites and axon terminals—and through attraction to different neural targets during axogenesis (figure *2b*). For example, in *Drosophila*, mutants can be generated that have different numbers of dendritic claws of a major cell class [62]. With cell number being constant, changes in dendritic claws influence odour-discrimination abilities due to increased odour selectivity. It remains unclear whether such variation represents a naturally viable route to evolutionary change given the widespread effects. However, comparisons of drosophilid olfactory circuitry illustrate that restructuring of circuits can happen through specific changes in cell morphology in upstream areas, i.e. changes in dendritic branch numbers and lengths [38]. Similarly, the relevance of specific food odours can be enhanced by either increasing the number of food odour encoding neurons or by increasing the number of connections they make to downstream relay neurons [69]. These examples illustrate how specific changes in projections, axons and dendrites can change circuits to accommodate behavioural change and innovation.

### 3.3. Circuit reconditioning

*Reconditioning* circuits involves the conservation of cellular anatomy and connectivity, but changes at the level of neuromodulation at synaptic sites caused by modifications in G-protein-coupled receptors, neuromodulators (i.e. -peptides or -transmitters) and other ion channel modifications [34,61,73,74]. By changing synaptic receptor content and cell physiology, valence and neural excitability can be modified, affecting circuit computations and behaviour. In this way, circuits can be anatomically identical between species but differ functionally [34,61].

Reconditioning is invoked in several instances of olfactory circuit evolution in dosophilids. In a comparison of courtship behaviour between *Drosophila melanogaster* and *Drosophila simulans*, peripheral and central circuitry are largely conserved in terms of cell number, but the central circuitry has diverged to initiate a different response to species-specific mating cues [39]. This involved retuning the response to a courtship pheromone from excitatory to inhibitory. Similarly, when examining four subspecies of *Drosophila mojavensis*, valence changes in central circuitry result in significant behavioural differences to a pheromone, despite conservation in circuit structure [75]. These examples illustrate that by keeping anatomy the same, pleiotropic costs of rewiring and functional constraints are avoided while functional changes can be enacted to accommodate behavioural change [61,76].

### 3.4. Overarching themes: evolvability and modularity

A critical reflection on the examples above is that many sources of neural variation, such as those discussed under restructuring and reconditioning, are unlikely to impact volumetric traits. Examples do exist where volumetric traits are an indicator of evolutionary changes in a circuit. For example, antennal lobe glomeruli are close proxies of underlying olfactory receptor function, and subspecialization of this system reflects olfactory adaptation [77]. Volumes of each glomerulus as well as glomeruli number thus closely mirror investment in synaptic numbers and receptor diversity, making volume and number accurate proxies [78–80]. However, even in this case, any investment in increased glomerulus size or overall number needs to be placed into the context of the overall olfactory circuit. Often, comparative analyses of brain size therefore likely concern those that specifically involve circuit expansion and replication. A key question is whether or not different behavioural subcircuits in a brain are equally likely to change through the three axes discussed above. The answer to this question depends on the specific functions of a circuit, as well as the developmental origin of its components. However, rather than expecting these axes to work independently of each other, we expect that the evolution of each circuit will be characterized along all three axes to differing degrees (figure *2c*). For example, the evolution of cerebellar nuclei is characterized by duplication, refinement and changes in the relative abundance of some cell groups [59], and in mormyrid fishes, changes in sensory perception pathways involve concomitant increases in cell numbers as well as lengthening and structural changes of axons [81].

Nevertheless, these three axes of neural variation are unlikely to have completely dependent roles in behavioural evolution. Their relative importance in the evolvability of a circuit likely depends on both functional constraints—where selection acts to conserve existing circuit functions—and developmental constraints—where developmental programmes influence the permissible range of trait variation [82]. Both ultimately affect the ability of a circuit to be rescaled. For example, we illustrate three ways in which this may play out:

#### 3.4.1. Modularity

A particularly prominent mechanism influencing the evolvability of behavioural subcircuits may be the degree of modularity [76]. Modularity is a characteristic of sensory and neuronal cells that lets them be subdivided into specific anatomical and functional units, defined by their shared developmental origin. This results in groups of neurons that are morphologically similar, and in close proximity to each other, forming modules.

#### 3.4.2. Spatial scale

A prominent factor affecting the evolvability of a circuit, and potentially in the occurrence of modularity, could be that any modifications of long-range projections are rare, while amplifications of short-range projections occur more often [3], favouring the evolution of modules [76]. This may suggest that modularity in circuit structure makes circuit motifs more susceptible to duplication, with a consequently higher chance of innovation.

#### 3.4.3. Information flow

Functionally, feedforward circuits (where information flows in one direction [60]) are potentially more likely to be modified than those with feedback connectivity, as any modification is less likely to disturb several other subcircuits and brain areas [61]. A deep characterization of circuits and identification of key circuit motifs is necessary to determine whether this assumption of evolvability of circuits holds true, but it is based on a logical assumption that with increased interconnectivity comes a higher potential for disturbance if one part of a circuit motif changes.

## 4. A multi-area integration circuit facilitating cognitive innovation

**Figure 3.**
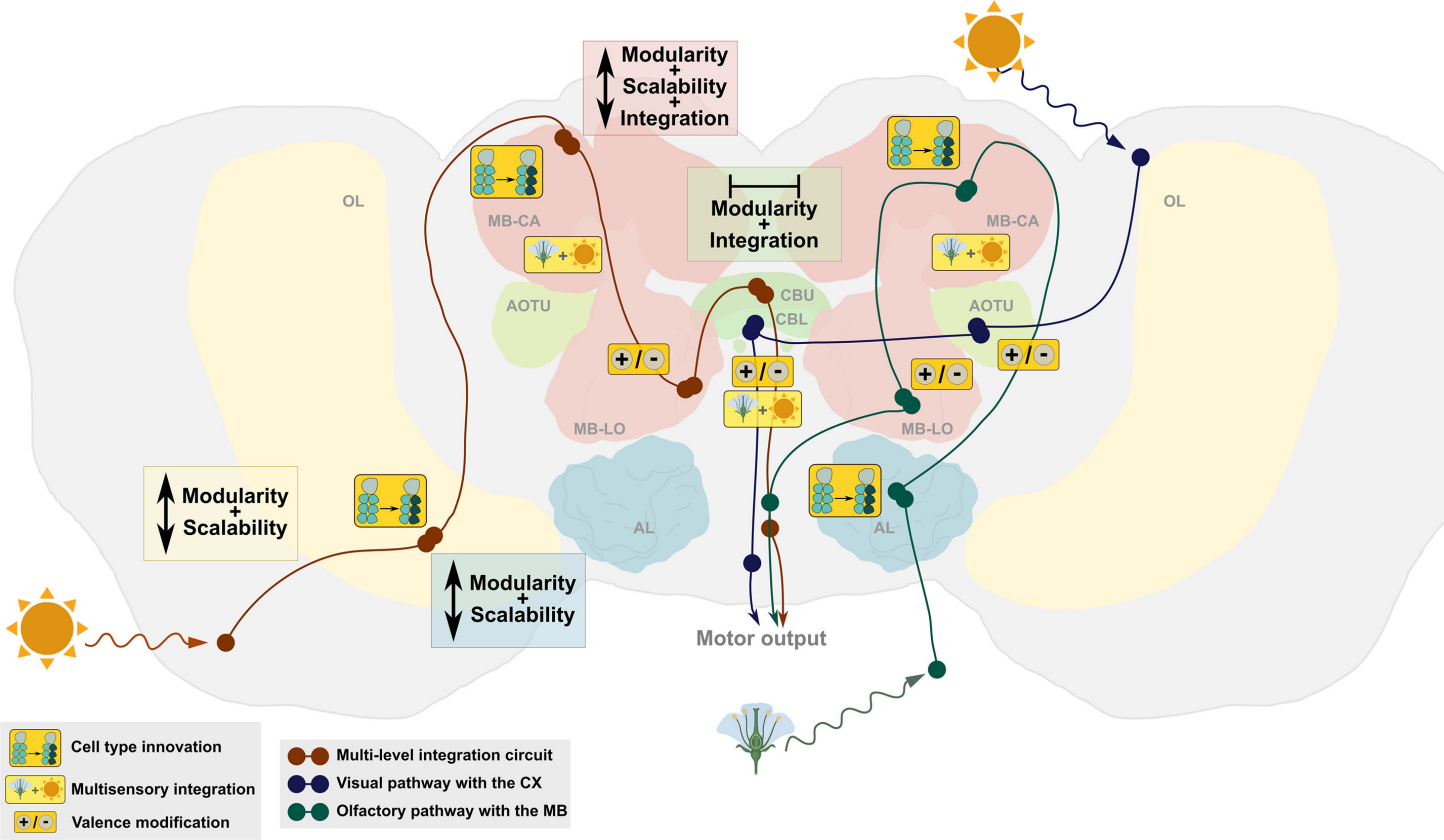
A circuit highlighting defining features and components of circuit-based cognitive evolution. Depicted are relevant brain structures and major pathways chosen to illustrate concepts of cognitive evolution. The circuit involves (i) visual stimuli, leading to the AOTU, to the lower division of the central body (CBL) and to descending neurons for motor output (blue); and (ii) olfactory stimuli leading from the antennal lobe to the mushroom body (MB) calyces, to the lobes and into other brain areas (e.g. lateral accessory complex). A multi-level integration circuit, where visual stimuli are received and decoded, lies within the MBs comprised Kenyon cells, where a signal is compared to past memory, and carried forward to the central complex where it is compared to heading direction, etc., to facilitate a motor output. Each part of these circuits shows different combinations of modularity, scalability and integration. In the central complex, we see a decoupling of modularity of cell types from the scalability of neuron numbers. Orange-boxed symbols indicate where different circuit changes can take place to modify circuit performance. Brain and neuropil shapes were redrawn from a *Heliconius doris* brain. Symbols were extracted from biorender.com. OL optic lobe, AL antennal lobe, MB mushroom bodies (LO lobes, CA calyx), AOTU anterior optic tubercle, CBU/CBL upper/lower division of the central body.

To illustrate how whole circuits may evolve to facilitate cognitive and behavioural innovation, and why this could be informative to the evolution of neural circuits and cognition generally, we showcase an example that connects the dimensions of circuit change to cognitive evolution. Specifically, we focus on an approximately eightfold expansion of neuronal cells and fourfold volumetric expansion in an integration centre, the mushroom bodies (figure 3), in a Neotropical tribe of butterflies, which occurs in the context of a highly conserved neural architecture [28]. This neural shift is linked to a behavioural innovation associated with spatially faithful foraging for pollen—a critical adult source of amino acids—over a prolonged lifespan, which necessitates an increased reliance on long-term memory [83,84].

### 4.1. Mushroom body circuits for integration of past and present

The mushroom bodies integrate sensory information and internal state to project changes in valence through learned and memorized contexts. Signal integration occurs where projection neurons from primary sensory areas connect to the internal cell types of the mushroom bodies, the Kenyon cells. Kenyon cells build localized and highly dense synaptic trees [85], connecting with six projection neurons on average in *Drosophila*, of which several need to fire for the Kenyon cell to be activated. Such global inhibition is required to have sparse but precise activation of cues [86–88]. Kenyon cells feedforward sensory signals through connections to other cells in distinct and localized patterns, within a region called mushroom body lobes. Distinct Kenyon cell types faithfully project and connect locally to downstream cells, forming lobe substructures named after the major Kenyon cell types: *α*, *β*, *α*′, *β*′ and *γ* lobes. The cells receiving information from Kenyon cells are critical for how mushroom bodies not only carry sensory information but also functionally integrate this with internal state. In particular, dopaminergic neurons (DANs) relate information about whether sensory information harbours a positive or negative association [89], and mushroom body output neurons (MBONs) relay DAN-modulated outputs of Kenyon cells to nearly all prominent brain areas in the insect brain [41].

How does this circuitry reflect behavioural adaptation and cognitive innovation? Essentially, information from sensory reception pathways is projected forward by Kenyon cells, compared to past experiences by DANs, and propagated by MBONs to cause a behavioural reaction. This circuit therefore allows individuals to adopt flexible behavioural responses in different conditions, be it in foraging, host-plant seeking or mate-choice [90–92]. It is quite remarkable how a seemingly simple circuit can contribute to a whole array of functions [93], thus making this site a particularly interesting case study in cognitive evolution. Indeed, this circuit motif seems to be evolutionarily widespread, and likely convergently evolved in vertebrate cerebella and mushroom bodies [41,93] (a deep genetic homology to the vertebrate pallium has also been proposed [94,95]). The make-up of feedforward organization, relatively short projections and the modular nature of Kenyon cells, make this circuit highly scalable, and indeed, across insects, the volumes of the calyces and lobes show massive divergence in size and shape, largely reflecting differing numbers of Kenyon cells [96,97]. Modification in DAN and MBON populations and projections are also likely but less broadly understood.

### 4.2. Mushroom body expansion in *Heliconius*

The high degree of variation in Kenyon cell number and mushroom body volume in *Heliconius* illustrates a number of different neural changes. First, expansion of the mushroom body circuit is likely driven by motif replication of Kenyon cells. Kenyon cell number is predicted to correlate with the capacity to store sparse information of sensory cues, providing ecologically relevant functional scalability [98]. Second, the circuit is restructured, with increased areas of synaptic innervation from visual projection neurons, and altered patterns of plasticity in synaptic pruning [28,83].

Shifts between the amount of olfactory or visual information integrated into the mushroom bodies are not uncommon [96,97], but the close phylogenetic proximity and ecological similarity of *Heliconius* and their outgroups provide a clearer view of the importance and specificity of this change. In *Heliconius*, the size of visual brain centres is largely conserved with other Heliconiini, meaning mushroom body expansion seemingly occurs in the context of conserved sensory perception. Currently, it is not known whether there is any increase in projection neurons, or a restructuring inside the calyx resulting in altered numbers of projection neurons each Kenyon cell connects with. Either scenario is fascinating, as a lack of concomitant shifts between sensory brain areas and areas that process this information is not necessarily intuitive. In addition, specific Kenyon cell classes, particularly *γ*, *α*, *β* and *β′* are thought to be expanded with concomitant changes in mushroom body lobe morphology, as well as increases in cell populations of the downstream circuit [99]. This illustrates that Kenyon cell expansion may target specific Kenyon cell populations, rather than global effects, which will be indicative of specific functions represented in particularly expanded mushroom body lobes. Indeed, *γ* lobes are generally associated with visual memory [100], which appears to be particularly important for these butterflies.

### 4.3. The cognitive circuit of mushroom bodies and central complex

The massive expansion in *Heliconius* butterfly mushroom bodies offers a compelling example where an isolated brain and behavioural phenotype occurs without wider concomitant shifts in behaviour and brain anatomy. However, the circuitry that involves the mushroom bodies operates in partnership with other brain regions, most notably another integration centre, the central complex. The central complex is a highly conserved brain area in all insects and has similar homologues in other arthropods [101]. Its layout and function can be largely explained by two cell types, columnar neurons, connecting the different parts of the central complex with each other, and tangential neurons connecting other brain areas with the central complex [102–104]. Its function *per se* is difficult to pin down but has been described as centred around four main tasks: representing an insects’ orientation in space by generating an internal signal, representations of goals, the selection of goals and directing a motor outcome [102]. At first, it is surprising to observe that central complex circuitry is so strictly conserved across large phylogenetic distances [105–108], but considering that so many circuits are integrated with the central complex, stabilizing selection caused by the constraint of conserving disparate functions must play a major role in its evolution, with interspecific divergence limited to (superficially) minor changes in anatomy [44,106].

Central complex functions overlap strongly with the cognitive processes that generate pollen-feeding behaviour in *Heliconius* butterflies, where a goal (the location of a pollen resource) is identified, learned and memorized [109]. Subsequently, sensory cues during navigation and foraging, such as optic flow, wind direction, polarized light and recognition of visual features, are integrated and compared with the original goal, and path integration allows the foraging route to be faithfully navigated [102,110–112].

Data from the *Drosophila melanogaster* connectome has identified significant direct and indirect connections between the mushroom body and central complex, illustrating that both integration centres are part of the same broader circuit to facilitate specific sets of behaviours [41,43]. Indeed, recent work has placed both brain areas into a common cognitive circuit [113,114] and both integrate multi-sensory information [115]. In *Heliconius,* this implies that learned resource locations are integrated with different visual information to cause a motor output related to feeding and navigation behaviour (figure 3). Essentially, a combined circuit including the mushroom bodies and central complex scales up the amount of integrated information to several sensory modalities and several instances of internal information, i.e. several dimensions of visual cues and olfactory cues, different valence cues indicative of past experiences through DANs, and goal and path integration cues to find target resources, such as flowers or an individual roost site.

The hard-wiring for this circuit (simplified in figure 3) has likely been in place throughout the whole tribe of Heliconiini, and at wider phylogenetic scales, but through an expansion of specific Kenyon cell types [99] and shifts in the relative importance of visual cues [28], as well as fine modifications in the neurotransmitter make-up of the central complex (Farnworth *et al.* [116]), the ancestral circuit is modified to allow the improved learning and memory performance necessary to drive behavioural innovation.

### 4.4. Insights into circuit evolvability

How does this particular circuit inform us about the evolvability of circuitry more generally? Mushroom body Kenyon cells and the circuit motifs they construct illustrate the importance of modularity and scalability in circuit evolution [93]. Their number can differ significantly even between closely related species, facilitated by developmentally modular production of a small number of cell types with a simple circuit structure. The central complex in contrast is modular, based on distinct repetitions of columnar neurons with stereotypic projections [102], but apparently not scalable, as inferred from its strict conservation. This implies a decoupling of scalability and modularity that illustrates the importance of the functional context of each sub-circuit. This also implies that a circuit, such as that shared by the mushroom bodies and central complex, will have specific sites and sub-circuit motifs that are more likely to be targeted by selection, such as the modular, feedforward and short-range projection circuit of Kenyon cells. These targets may be motifs that are modifiable without disruption of ancestral circuits, while other sites cannot be modified in the same, replicative, manner. Instead, in these more constrained conditions, we may expect a more dominant role of minor circuit restructuring or reconditioning. Importantly, only changes in replication are detectable at a volumetric level, creating a bias in how prominent certain axes of neural change are perceived to be. As such, these volumetric shifts are unlikely to reflect the full breadth at which selection acts across circuits underpinning behavioural variation.

## 5. Future trends in comparative cognition and neural circuit evolution

We have focused above on a particularly striking case study of natural variation in brains and behaviour. Whether or not the insights invoked from this case study generalize remains to be seen. Indeed, a deep understanding of evolutionary processes can only be achieved by developing in-depth case studies across a diverse range of species [32]. Here, we highlight four major questions that will need to be addressed to further the understanding of the evolution of neural circuitry and cognition.

### 5.1. How independent are axes of neural change?

We have illustrated that three major axes of neural change—replication, restructuring and reconditioning—are not mutually exclusive. However, do different ancestral circuits and functions place specific scenarios on divergent circuits such that these axes are independent to differing degrees? For example, to what extent is adding more cells generally sufficient to enact functional modification? What else needs to be adjusted and how do these factors depend on ancestral circuitry and function?

### 5.2. Do different axes of neural variation have distinct costs?

Energy expenditure is the most consistent constraint on neural circuit change [19,21,22]. However, how does this play out in each specific circuit? Does this act against circuit replication? And is modularity, involving short-range projections in a high number of cells, combined with long-range projections in a few, a response to selection for energy efficiency or something exploited by it? And are such concepts applicable across the animal phylogeny or do we see differences in inherent properties of arthropod and vertebrate brains?

### 5.3. What defines functional constraints acting on a circuit?

While we have a clearer concept of what developmental constraints are, we are much less clear on the degree to which circuits are constrained by current functions or involvement in multiple interdependent behavioural pathways. How does selection navigate functional integration? How do you change a circuit in one context without disturbing it more broadly? And, if some circuits are changeable through scaling, what makes them so?

### 5.4. What aspects of behavioural variation are biased towards different axes of neural variation?

Are learning/memory improvements replication focused, while increased motor control includes restructuring or replication? Or does the diversity of neural circuits within a species allow for substantial change in behavioural performance by co-opting existing circuits? Do changes in cellular responses to cues, through neural reconditioning, have restrictive effects on motivation or attraction, or can they also affect how information is integrated to support behavioural novelty?

Understanding the neural basis of cognitive evolution to the point of answering all these questions will inevitably be challenging. This is in part due to practical concerns, including the tractability of neural circuits and development of tools that enable a greater range of species to be included in comparative studies. However, as we broaden the phylogenetic sampling that can fully employ the technological advances now at our grasp, be it connectomics or gene modification, this is a fitting time to examine the evolution of neural circuitry and cognition.

## Data Availability

This article has no additional data.
